# Biomaterial composed of chitosan, riboflavin, and hydroxyapatite for bone tissue regeneration

**DOI:** 10.1038/s41598-023-44225-0

**Published:** 2023-10-09

**Authors:** Justyna Gaweł, Justyna Milan, Jacek Żebrowski, Dariusz Płoch, Ireneusz Stefaniuk, Małgorzata Kus-Liśkiewicz

**Affiliations:** 1 Institute of Biotechnology, College of Natural Sciences, University of Rzeszow, Pigonia 1 St, 35‑310 Rzeszow, Poland; 2 Laboratory of Innovative Toxicological Research and Analyses, Institute of Medical Studies, Medical College, Rzeszów University, Aleja Majora W. Kopisto 2a, 35-959 Rzeszow, Poland; 3 Institute of Materials Engineering, College of Natural Sciences, University of Rzeszow, Pigonia 1 St, 35‑310 Rzeszow, Poland

**Keywords:** Biotechnology, Medical research, Materials science

## Abstract

Biomaterial engineering approaches involve using a combination of miscellaneous bioactive molecules which may promote cell proliferation and, thus, form a scaffold with the environment that favors the regeneration process. Chitosan, a naturally occurring biodegradable polymer, possess some essential features, i.e., biodegradability, biocompatibility, and in the solid phase good porosity, which may contribute to promote cell adhesion. Moreover, doping of the materials with other biocompounds will create a unique and multifunctional scaffold that will be useful in regenerative medicine. This study is focused on the manufacturing and characterization of composite materials based on chitosan, hydroxyapatite, and riboflavin. The resulting films were fabricated by the casting/solvent evaporation method. Morphological and spectroscopy analyses of the films revealed a porous structure and an interconnection between chitosan and apatite. The composite material showed an inhibitory effect on *Staphylococcus aureus* and exhibited higher antioxidant activity compared to pure chitosan. In vitro studies on riboflavin showed increased cell proliferation and migration of fibroblasts and osteosarcoma cells, thus demonstrating their potential for bone tissue engineering applications.

## Introduction

Bone naturally possesses the intrinsic ability to regenerate during skeleton development or remodeling throughout adult life, but also in response to various types of injuries^1^. In comparison to soft tissues, which heal predominantly through scar tissue formation, bones heal through the generation of new bone. Therefore, bone repair can be described as a regenerative process^2,3^. In the clinical settings, the most common form of bone regeneration is fracture healing^3^. The biology of bone healing is very complex, involving many types of cells and intracellular and extracellular molecular signaling pathways to regenerate the skeleton and restore its function. It comprises a well-orchestrated series of events; including the formation of hematomas, the formation of fibrocartilaginous callus, the formation of bone callus, and bone remodeling^4^. However, there are cases of bone fractures in which bone regeneration is impaired; there may be delayed union or non-union of the bones. This is due to major injuries, infections, certain diseases such as osteoporosis, or other surgical interventions that led to the excision of the bone. In such cases, a bone graft is essential^5^. Nowadays, many methods are available, including allograft, autograft, and synthetic bone grafts. The autograft is harvested from the patient’s own body from a different unaffected site, while the allograft is from living donors or cadavers. Autogenous bone grafts remain the gold standard therapy, since the donor and recipient are the same individuals and there are no issues with histocompatibility. The allograft is the next best alternative; however, the processing step may alter mechanical competency and there is a risk of transmitting disease. Therefore, significant research and development has been done to generate alternative options in the form of synthetic bone substitutes^6,7^.

The frequency of bone grafting is, in fact, the second most frequent tissue transplant worldwide, occurring immediately after a blood transfusion^8^. Half a million patients are reported to receive bone defect repairs per year in the United States and Europe with an estimated cost exceeding US$ 3 billion^9^. It is estimated that the number of people at high risk of osteoporotic fractures will double by 2040 in the Western world due to demographic aging; therefore, there is a high demand for the development of new types of synthetic bone graft^10^, especially a graft that combines a synthetic scaffold with diverse biologic elements to stimulate cell infiltration and new bone formation^6^.

Biodegradable materials have been at the forefront of cutting-edge research and offer a truly viable option in the design and manufacture of composites in biomedical engineering^11^. In the treatment of bone defects, scaffolds made of biodegradable materials can provide a bridge for the growth of new bone tissue in the gap. It may be a platform for cells and growth factors, which will eventually be degraded and absorbed in the body and replaced by new bone tissue. In general, the objective of tissue engineering is to allow the body's own cells to replace the implanted scaffold over time. Thus, biodegradability is one of the most important features^12^. Numerous synthetic and natural polymers have been used in the attempt to fabricate scaffolds. Commonly used synthetic polymers, including polystyrene, poly-l-lactic acid, polyurethane, and polyglycolic acid, can be easily fabricated with a tailored architecture^13^. However, these materials are at risk of rejection and are not considered bioactive. Contrary, the natural polymers used to manufacture scaffold, such as collagen/gelatin, alginate, chitosan, silk, hyaluronic acid, elastin^14^, are biologically active, have low risk of rejection, and usually promote cell proliferation. On the other hand, the main drawback of this polymer is that they have relatively poor mechanical properties, which limits their application. Therefore, the development of composite scaffolds that consisting of several phases is desired because it may combine the good processability of the composite with the excellent mechanical strength^12,15^.

Chitosan (CS) is a polycationic aminopolysaccharide obtained by N-deacetylation of chitin, the main ingredient of crustacean shells, fungi, or insects^16^. It is a well-known biocompatibility, biodegradability, and cytocompatibility component of various scaffolds and composites. Due to its excellent film-forming properties, many biomedical applications have been considered^16–18^. Among them, the biomaterial composed of chitosan and hydroxyapatite seems to be one of the main types of materials considered for bone regeneration^19^. Hydroxyapatite (HAP) is the main mineral component of bone tissue, so it has been widely used, such as bone tissue engineering or skeleton implants^20,21^. Furthermore, the chitosan/HAP scaffold has been shown to act as a proliferative support for bone cells^22^. Riboflavin is one of the vitamins necessary in the human diet. It can be provided primarily from plant sources^23^. In industry, it is most often produced synthetically, while it is also synthesized by various types of microorganisms^24^. The endogenous riboflavin synthesis pathway may be the target of the development of antimicrobials and exogenous riboflavin against infectious diseases caused by microorganisms. Vitamin B2 is used for the prevention and treatment of migraines^25^, it has excellent antioxidant properties, which is why the animal body needs it to protect against oxidative stress^26^. In nanotechnology, riboflavin is used for targeted drug delivery, in optoelectronics or biosensors^27^. Riboflavin is also being investigated for applications in photodynamic therapy^28,29^. Moreover, a potential use of riboflavin as adjuvants to improve bone metabolism is also suggested^30^.

In the past few decades, many techniques have been used to prepare scaffolds for tissue repairs, such as thermally induced phase separation, freeze-drying, gas foaming, electrospinning, 3D printing, solvent casting, and phase separation^31–34^. Each of the methods has its own advantages and disadvantages. Among them, the casting and drying technique is commonly used because this is a simple, low-cost method and there is no need for any sophisticated apparatus. No matter the choice of the manufacturing assay, the scaffold has to exhibit specific features, depending on their future application. In particular, when composites are considered, the successful incorporation of the component into the matrix is anticipated. He et al. reported on the casting/evaporating method of composite synthesis^35^. Indeed, they obtain hybrid films where the nanoparticles of hydroxypaptite were uniformly dispersed in the NaOH/urea aqueous system. Other studies show that collagen films, synthesized by casting aqueous collagen solution with solvent, may serve as cell carriers suitable for tissue engineering^36^.

**Figure 1:**
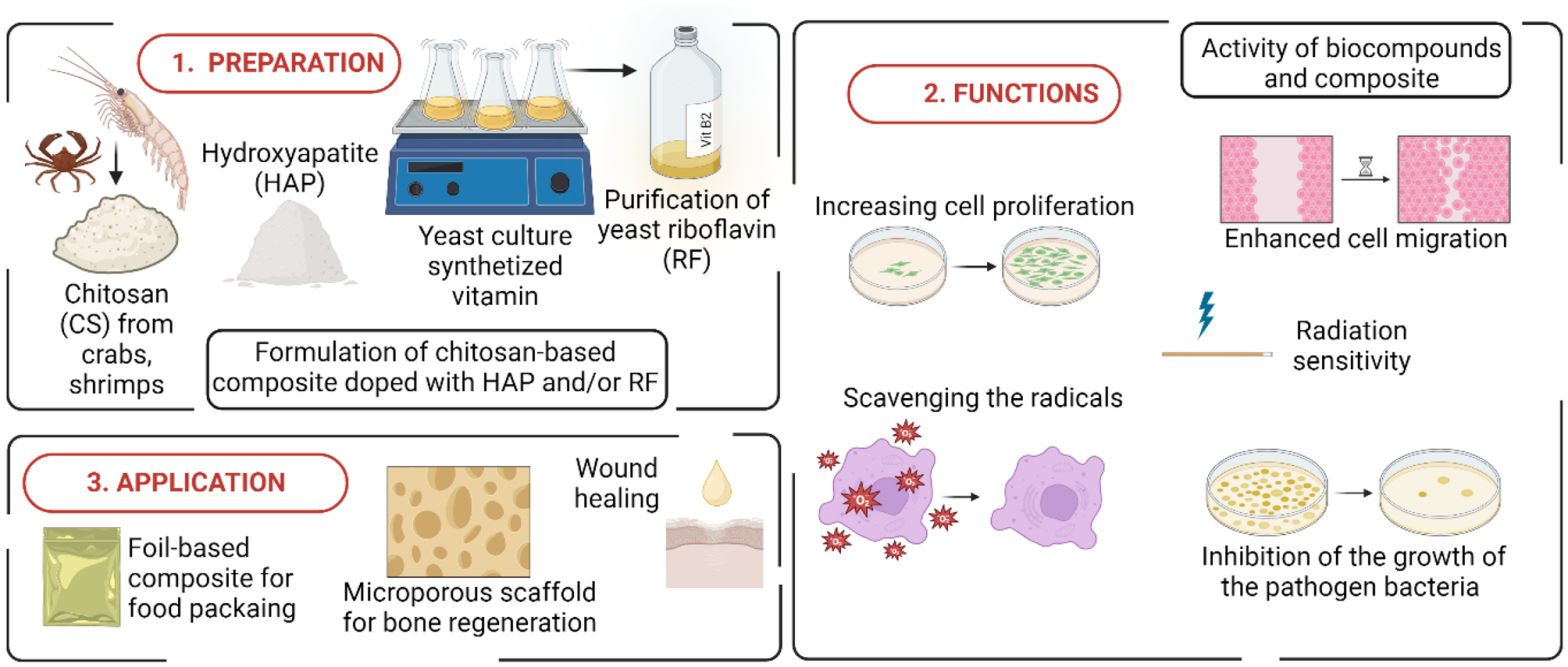
Scheme of the procedures for composites synthesis and the subsequent steps in their functions and evaluation of applications.

In this study, chitosan-based composites, incorporated with riboflavin and hydroxyapatite, were fabricated and their properties were evaluated (Fig. 1). The physical, structural, and biological properties of the nanocomposite film and riboflavin, as the naturally occurring ingredient, were characterized. For the first time, the composite was performed with the riboflavin obtained and purified from yeast, thus the film exhibits a high rate of bioavailability. Usually, the ABTS or DPPH assay is the most commonly used method for measuring antioxidant activity; however, the most efficient electron paramagnetic resonance (EPR) spectroscopy is considered. Therefore, here the spin trapping technique in combination with EPR spectroscopy was used to demonstrate the generation of hydroxyl radicals by RFirradiation. Raman analysis showed that there is an interlinkage between chitosan and HAP, whereas there is no interlinkage with RF. This may cause leakage of the RF from the composite and make it bioavailable. To estimate the impact of RF, extracted riboflavin was tested on eukaryotic cells to assess its cytotoxicity and proliferative potential. In addition, the antioxidant and antibacterial capacity of these films was assessed. Our hypothesis is that the combination of naturally occurring chitosan and riboflavin with hydroxyapatite results in a material with unique characteristics that can be used further as bone regeneration material.

## Materials and methods

### Materials

For composite preparation, medium molecular weight chitosan (190–310 kDa), aqueous solutions of acetic acid (99.8%) and hydroxyapatite were purchased from Sigma-Aldrich (USA). Synthetic riboflavin (Sigma-Aldrich) was used as a control for the RF naturally obtained. For the antioxidant activity of the compound, ABTS (Pol-Aura, Poland) was used. 3-(4,5-dimethylthiazol-2-yl)-2,5-diphenyltetrazolium bromide thiazolyl blue tetrazolium bromide (MTT) and dimethylsulfoxide for the characterization of yeast riboflavin, were purchased from Sigma-Aldrich (USA) and Stanlab (Poland), respectively. For EPR measurements, potassium hydroxide and hydrogen peroxide (Chempur, Poland) as well as α-(4-pyridyl-1-oxide)-N-tert-butylnitrone (Sigma-Aldrich, USA) were used.

### Cell lines, strain and culture conditions

Cell lines and yeast strain were obtained from the University of Rzeszow Institute of Biotechnology collection. For the cell culture experiment, Dulbecco’s Modified Eagle Medium High Glucose (DMEM, Corning, USA), fetal bovine serum (FBS, Biowest, France), phosphate buffered saline w/o magnesium and calcium (PBS, Corning, USA), 0.25% trypsin (PAA, Germany), antibiotic solution (Antibiotic Antimycotic Solution, Sigma-Aldrich, USA) were used. No human or animal was involved in the study. Human bone osteosarcoma epithelial cells (U2OS) and mouse fibroblasts (NIH3T3) were used. Standard techniques were used for mammalian cell cultures. Cells were cultured in DMEM medium supplemented with 1% antibiotic solution and 10% fetal bovine serum at 37°C in an atmosphere containing 5% CO_2_; subculture with typical trypsin treatment. Through the study, the engineered strain *Candida famata* (AF-4/SEF1/RIB1/RIB7) was used. YPD medium (BTL, Poland) containing yeast extract (10 g/L), peptone (10 g/L) and dextrose (20 g/L) was used to prepare the precultures.

### Composite preparation and characterization

The chitosan-acetic acid solution was prepared as described elsewhere^37^. Composites, in the form of films, were obtained by using the casting/solvent evaporation method. To prepare the composites, 7.5 mL of 2% chitosan-acetic acid solution was taken and mixed with 3.5 mL of riboflavin (60 µg/mL) and 5 mL of 1% hydroxyapatite. To simplify further sample nomenclature, an abbreviation was used, where CH stands for chitosan film, CH/RF stands for chitosan mixed with riboflavin, and CH/RF/HAP stands for chitosan mixed with riboflavin and hydroxyapatite. To obtain the solid form of the film, 10 mL of each liquid composite was poured onto the teflon surface and dried at 22 °C for 48 h. 

### Composite surface morphology examination by scanning electron microscope (SEM)

The composite materials in liquid form were placed on the aluminium grid and dried at room temperature for 24 h. Prior to SEM observation, the samples were sputtered with a 4 nm gold layer to provide them with electronic conductivity and avoid electronic charging during SEM imaging. SEM analysis was carried out using a TLD electron detector in secondary electron (SE) mode with the following operating parameters: voltage 3 kV, sample current 50 pA, and magnification 1–50 kx.

### Thickness measurements

3D scans with foil thickness measurement were performed on an OLYMPUS LEXT OLS5000 3D confocal laser microscope with a 10× objective (MPLFLN10xLEXT). The samples were attached to double-sided adhesive tape, which eliminated the problem of rolling thin foils. The average thickness of the foil was measured using the "step height" function, taking the plane of the adhesive tape surface as the base value—"0", and the plane of the glued sample within the field of view of the lens—as the value of the sample thickness, omitting the area directly at the edge. The field of view of the lens was: 1280 × 1280 µm.

### FTIR-ATR analysis

Infrared spectral analysis of riboflavin, chitosan, and chitosan-riboflavin-hydroxyapatite composites was performed using IRSpirit-T (Shimadzu, Japan) spectrometer with attenuated total reflectance (ATR) accessory (QATR-S) at a resolution of 4 cm^−1^ and 64 scans. The surface of the diamond crystal was cleaned with 70% ethanol to remove residuals from previous samples and a background was collected prior to each measurement. The spectra were baseline corrected and the area normalized before the analysis using the ChemoSpec package^38^ in the R programming language^39^.

### Antioxidant properties of composite

The method for determining the scavenging capacity of ABTS free radicals was based on the method described above^40^, with some modifications. Briefly, equal amounts of liquid forms of composites were mixed with ABTS (7 mM) and kept dark for 10–30 min. The samples were centrifuged (5000×*g*, 15 min) and the absorbance of the supernatants was measured at 734 nm. The activity of scavenging (*SA%*) radicals was expressed as follows:

$$SA\%=\frac{{Abs}_{ctrl}-{Abs}_{sample}}{{Abs}_{ctrl}}\times 100$$

where Abs_ctrl_ and Abs_sample_ are the absorbances of the control sample and the tested sample, respectively. 

### Antibacterial activity

For the antimicrobial assay, the gram-negative strain *Staphylococcus aureus* (ATCC 25923) was employed. Nutrient broth and agar (BTL, Poland) or phosphate buffered saline (Sigma-Aldrich, USA) were used for cell culture and serial dilutions, respectively. All assays were carried out in a laminar flow hood (Thermo Scientific, MSC Advantage). The bacteria preculture was overnight incubated under aerobic conditions at 37 °C, and then diluted to give an initial concentration of approximately 1 × 10^6^ cells/mL. Aliquots of composite material (CS/HAP/RF) were mixed with inoculum and incubated for another 24 h with shaking. After this time, the aliquots of the control and tested samples were serially diluted and poured onto the agar plate. The number of colony-forming units (CFU) was counted and expressed as the CFU/mL. All experiments were carried out in triplicate.

### Riboflavin characterization

#### Spectrum identification

*Candida famata* was grown for 5 days in YNB medium composed of yeast nitrogen base (1.7 g/L), glucose (10 g/L) and glycine (10 g/L) at 30 °C on a rotating shaker (150 rpm) in the dark. The biomass was sediment after centrifugation (10,000×*g*, 15 min) and the supernatant was filtered through a 0.22 μm syringe filter (Merck). As reference riboflavin, the standard of vitamin B2 purchased from Sigma-Aldrich was used. The absorption spectrum and fluorescence emission spectrum of isolated and reference RFs were recorded (Infinite M200, Thermo Scientific).

#### EPR measurements of radicals

Superoxide anions were generated immediately before the EPR measurements by mixing 50 µL KOH solution (25 mM) with 50 µL hydrogen peroxide (25 mM) and then 50 µL of DMSO and 50 µL of POBN (180 mM) were added. After 5 min, 100 µL of solution of isolated RF (60 µg/mL) and 100 µL of POBN solution (prepared as above) were mixed and placed in a quartz glass test tube on the BRUKER FT-EPR ELEXSYS E580 spectrometer (BRUKER BIOSPIN, Billerica, MA, USA) with digital registration. The following settings were used: frequency, 9.403467 GHz; central field, 348.50 G; modulation amplitude, 0.5 G; modulation frequency, 100 kHz; microwave power, 47.43 mW; power attenuation 5 dB; scan range, 100 G; conversion time, 30 ms; sweep time, 30.72 s. The EPR spectra were recorded and analyzed using Xepr 2.6b.74 software. Xepr is a comprehensive software package of the ELEXSYS series, accommodating the needs of every user with highly developed acquisition and processing tools.

#### Raman spectroscopy

The identification of RF in the extract of yeast was performed by comparing the Raman spectra of the extract and the reference RF sample in solution and in the form of a powder. The i-Raman Plus (B&W Tek, Delaware, USA) spectrometer with a 785 nm diode laser as the excitation source and CCD cooled detector was utilized. The instrument was coupled to a video microscope (B&W Tek Inc., BAC151, 20× objective lens). The spectra were collected at 5 accumulations, acquisition time 15 s, and laser power of at least 20 mW. The sample solution was deposited on the aluminium folia and allowed to evaporate before measurements.

#### Biological properties of yeast riboflavin

Metabolic activity and cell migration ability after RF treatment were performed using the MTT and wound healing assay, respectively^41,42^. Cells were seeded onto the 96- or 12-well plate at a density 3 × 10^4^ or 1 × 10^5^ cell/well. For the MTT assay, 200 µL of DMEM containing different concentrations of RF was added to the wells. After 24 h of incubation, the medium was discarded and then 200 µL of MTT (0.5 mg/mL) suspended in DMEM was added per well. After 4 h of additional incubation, the supernatant was discarded again and the purple formazans were dissolved with 100 µL of DMSO. The absorbance was read at a test wavelength of 570 nm and a reference wavelength of 630 nm and the OD results were presented as the percentage of controls values. For the wound healing assay, confluent cells in a 12-well plate were scratched in the diameter of the culture well. The cells were then washed with PBS to remove non-adherent and dead cells. The wells were filled with 500 µL of DMEM supplemented with various concentrations of RF. Wound closures were periodically visualized (0, 24, and 48 h) under a microscope and compared with etoposide treatment (a well-known inhibitor of cell migration).

## Results and discussion

### Biomaterial’s morphology

**Figure 2:**
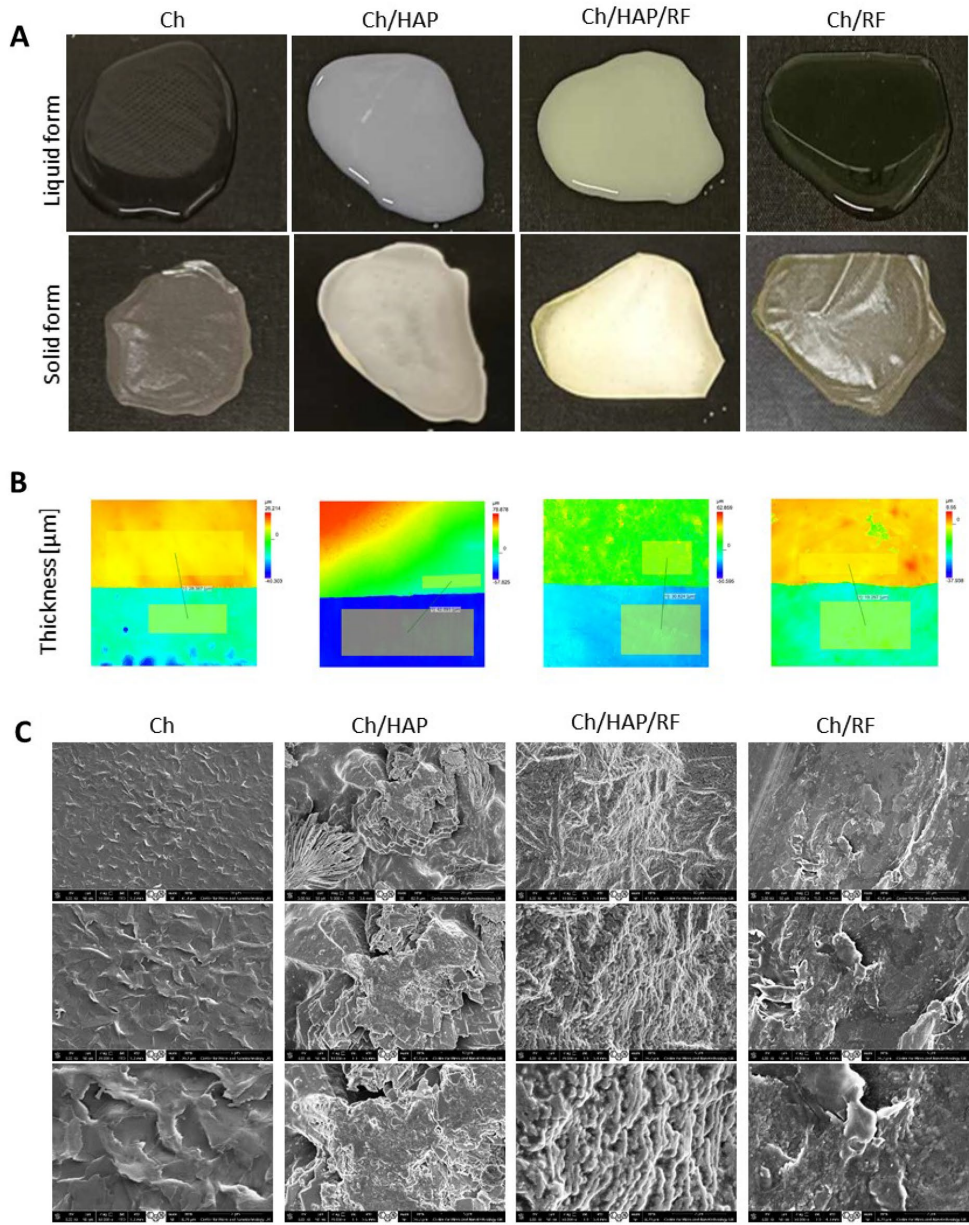
Representative film images (**A**) with thickness estimation (**B**), and SEM micrographs (**C**) of the pure chitosan and composite material doped with hydroxyapatite and/or riboflavin.

Self-healing of the bone from damage caused by infection or trauma is limited; therefore, external intervention is needed to stimulate bone repair^43^. At present, various biomaterials are being designed to develop a composite material suitable for bone regeneration. Hydroxyapatite is often used in dental and orthopedic implants, because it can induce the formation of bone-like apatite and promote bone healing^44^. Fillers such as bioactive nano-hydroxyapatite are also used, where the nano-hydroxyapatite surface allows osteoblastic cell adhesion and growth; therefore, new bone is formed by substitution from adjacent normal bone^43,45^. However, HAP possess some limitations, that is, its tendency to fragment and trigger inflammatory reactions^22^. Hybrid materials composed of chitosan and hydroxyapatite have been reported to have synergetic actions to significantly improve the biocompatibility and osteoconductivity in living bodies of these materials^46^. In view of the biocompatibility requirements of bone regeneration biomaterials, hybrid composites based on chitosan and hydroxyapatite were fabricated. Moreover, in order to develop a more efficient biomaterial, doping it with riboflavin as the proliferating agent could be of interest and desirable. The casting/solvent evaporation method was used to manufacture the nanocomposite, in which the solvent is removed by standard drying practices (Fig. 2A). This technique does not require complex instrumentation, harsh chemicals, or high temperatures; thus, it is a simple, cost- and time-effective assay to produce a chitosan-based film^47^. In addition, they are easy to handle since in the solid form they do not require specific storage, i.e. low temperature or humidity. The thickness of the film was estimated using 3D confocal laser microscope (Fig. 2B). The manufacture of chitosan-based composite results in films with thickness of 28, 19, 42, and 30 µm for Ch, Ch/RF, Ch/HAP, and Ch/HAP/RF respectively. This analysis demonstrated the difference between the thickness of films doped with riboflavin, as these composites were thinner. Indeed, decrease in the thickness of the chitosan-based films occurred after doping it with various organic compounds^48^. Therefore, modification of chitosan-based scaffolds provide the ability to tailor the physiochemical properties of the composites. The surface morphology of the composite materials was visualized by SEM (Fig. 2C). The SEM images revealed that the surface of the composite materials is porosity and contains pores, compared to the smooth surface of pure chitosan. These characteristics may contribute to the cell adhesion of bone regeneration material, due to the phenomenon that porosity may play an important role in osteoconductivity^49^. Some studies have demonstrated a greater degree and faster rate of bone ingrowth or apposition with percentage porosity. Sufficient porosity of suitable size and interconnections between pores provides an environment to promote cell infiltration, migration, vascularisation, nutrient and oxygen flow, and removal of waste materials while being able to withstand external loading stresses^50,51^.

### Biomaterial’s chemical structure

**Figure 3:**
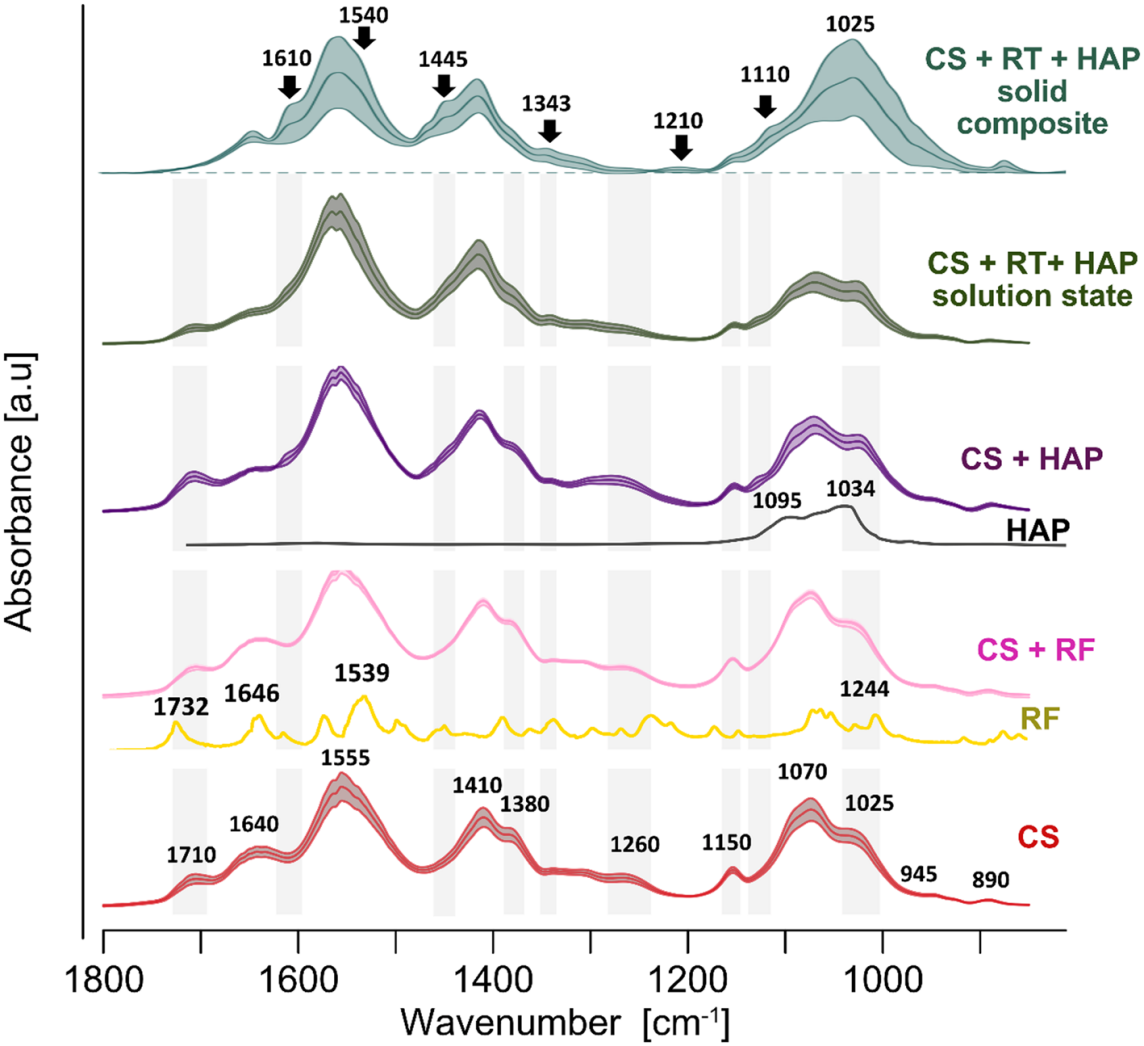
ATR-FTIR spectra of chitosan (CS)(lower), riboflavin (RF), CS-RF, hydroxyapatite (HAP), CS-HAP, and the chitosan-riboflavin-hydroxyapatite CS-RF-HAP as mixture in solution and as solid composite (upper) (from bottom to top). The mMeans and SD of the absorbances are presented as mid-darker lines and shaded ribbons, respectively; n = 15. Relevant for discussion regions are shaded grey and bands are labelled.

We further used vibration spectroscopy to identify the chemical composition of yeast extract prepared for the experiments and to inspect chemical structure and thus possible interlinks between compounds/phases comprising the CS-RF-HAP composite (Fig. 3).

Chitosan was characterized by major bands around 1640 cm^−1^ (amide I, C=O stretch), 1555 cm^−1^ (amide II, N–H bend), 1410 cm^−1^ (C–N stretch), 1380 cm^−1^ (CH_3_ deformation), 1150 cm^−1^ (C–N stretch, C–O–C stretch), 1070 cm^−1^ (C–O–C stretch), 1025 cm^−1^ (C–O–C stretch), most of which are consistent with reports from other studies^52–55^. Infrared spectroscopy showed substantially altered spectrum of the CS/HAP/RF composite compared to that of pure chitosan. This reflected not only the presence of added HAP, enriching spectrum with bands assigned primarily to vibrations of the phosphate group (strong bands located in the region between 1150 and 1000 cm^−1^^56–58^, but also showed changes in the location, intensity, and shape of bands attributed to CS. The most striking changes were related to the amide I band, which corresponds to the C=O stretching vibrations of the residual N-acetylated groups, and to the amine and hydroxyl groups evenly distributed along the chitosan chain. These groups serving putatively as interactive sides were deduced on the basis of disappearance or diminishing intensity of bands at about 1640, 1308, 1150 cm^−1^, assignation of which was discussed in the literature^59–63^.

To have deeper insight into mechanism of the interlinkage formation between compounds of the composite we additionally analysed the spectra of chitosan-riboflavin, chitosan-hydroxyapatite, and all the composite compounds (CS-RF-HAP) in fluid state (Fig. 3). We took into consideration also reference spectra of the pure riboflavin powder and HAP. The RF is characterized mainly by the C=O stretching vibrations at 1732 and 1648 cm^−1^, the major peak at around 1540 cm^−1^ from the C–C stretching vibrations of the ring I coupled with the CN double bond stretching as well as by multiple bands attributed to the C–C, C–N stretching and the CH or CNH bending vibrations^64^.Addition of riboflavin to chitosan did not alter visibly the spectral features of chitosan, indicating that majority of functional groups of the chitosan remained available for potential cross-linking between the chitosan and HAP to form a scaffold. Indeed, the presence of HAP reduced the intensity of the CS bands at 1640 cm^−1^ and 1150 cm^−1^ as well as the ratio of the absorbance intensities I_1070_/I_1025_. Some decrease of the absorbance was also observed at 1380 cm^−1^. Further addition of the riboflavin slightly increased the effects, that may indicate some contribution of RF formation of the cross-linkages. However, the mixture of all the compounds in form of solution did not mimic completely the spectrum of CS-RF-HAP composite in solid phase, although all the measurements were conducted for the air-dried samples. This indicated that more abundant cross-linkage is established during formation of the composite than in the course of drying the mixture of the compounds in solution.

Earlier studies on composites produced on the basis of CS and HAP showed similar spectral characteristics that were mainly attributed to the formation of hydrogen bonds between CS and HAP through the functional groups mentioned above^56,62,63,65,66^. The examined spectra also resembled, to some extent, phosphorylated chitosan spectra^67,68^, which may also suggest the formation of covalent bonds with the participation of phosphate groups. It should be mentioned that the FTIR-ATR technique, used in this study, reflects chemical structure only on the surface of examined samples. The obtained spectral features indicate also some heterogeneity of the composite sheets in terms of the compounds ratios and interlinks formed, which was demonstrated by a relatively high standard deviation of the spectral absorbances. (Fig. 3). This study did not provide clear evidence that RF was bound via chemical bonds to the CS-HAP scaffold, as RF contribution was observed in the composite spectrum as a very weak bands at 1740 cm^−1^, at 1650 cm^−1^ (a small sharp peak instead of reduced broad amide I), and possibly contributing to the shoulder at 1540 cm^−1^ (frequency similar to that of the major RF band), all without substantial location changes compared to the reference RF spectra^69,70^. Possibly, this compound is just adsorbed, and thus is easily available to perform its biological activity. Infrared spectroscopy also allowes us to detect carbonation of HAP (a shoulder at 1443 cm^−1^ and a weak band at 870 cm^−1^), which made the composite matrix of less regular structure compared to HAP without substitution^71^. This may alter the physical and chemical properties of the scaffold^72^ in order to better perform in potential regenerative applications, particularly when the matrix is supplemented with RF.

### Characterization of composites

**Figure 4:**
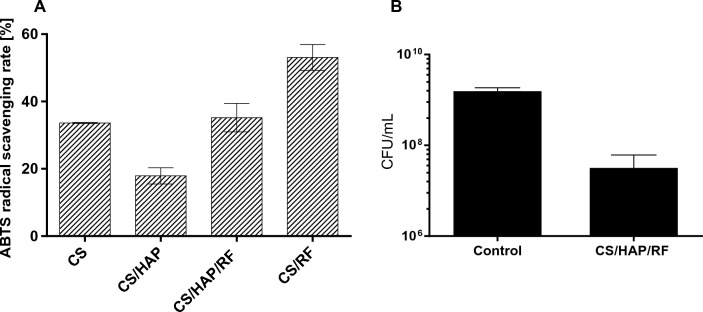
The ability of pure and doped-composites to scavenge ABTS free radicals (**A**). The antibacterial activity of composite material (CS/HAP/RF) compare to the control group, non-treated bacteria (**B**). The error bars indicated the standard deviation of three independent experiments.

The development of new types of antioxidant biomaterials is of utmost importance due to the increasing problems with pathogen transmission. Hence, in the next study the antioxidant and antimicrobial potential of the active biomaterial was examined. In general, the incorporation of RF increased antioxidant activity compared to pure and hydroxyapatite–doped chitosan (Fig. 4A). Numerous studies have investigated the antioxidant properties of some vitamins such as vitamin E, vitamin C, and carotenoids (review in^26^) and their effects on human health. However, riboflavin is the one neglected antioxidant vitamin, which in fact acts as a coenzyme for redox enzymes in the forms FAD and FMN. The results of the reviewed studies indicate that the antioxidant nature of RF is due to protection of the body against oxidative stress, especially lipid peroxidation and reperfusion oxidative^26^. Researchers have already shown the development of the biodegradable material incorporated with riboflavin, however its antioxidant potential has not been evaluated^73^. The antimicrobial results showed the inhibitory effect on *Staphylococcus aureus*; the number of colony-forming units was significantly reduced (Fig. 4B). Thus, the results of this work are very valuable in the field of using material to bone regeneration and impact on the oxidative injuries, as well as the material with enhancement of antibacterial potential, i.e. in food packaging industry.

This antibacterial performance of the riboflavin-doped composite may be the result of the photosensitizer action of RF. Irradiated riboflavin is known togenerate significant intracellular ROS and induce oxidative stress^74^. Thus, we further examined the nature of the RF isolated from yeast, which was the component of the composite materials.

### Physico-chemical and biological properties of the riboflavin

**Figure 5:**
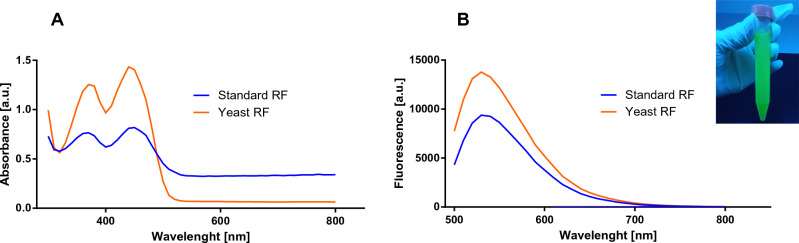
UV–vis (**A**) and fluorescence emission spectrum (**B**) of standard riboflavin sample and isolated riboflavin after 5 days of incubation. The inset with the fluorescence of yeast RF under UV irradiation.

Riboflavin production was determined, during a fermentation period that lasted 5 days. During this experiment, we noticed that the color of the fermentation broth gradually turned yellow and emitted fluorescence when exposed to UV light (Fig. 5 inset). To characterize riboflavin, its ultraviolet–visible and excitation/emission spectra were recorded with a fluorescence spectrophotometer. The absorption spectrum and fluorescence emission spectrum of isolated and reference riboflavin are presented in Fig. 5A and B, respectively. In the wavelength range of 300 to 800 nm, the absorption spectrum of both riboflavin demonstrated two absorption peaks at 370 and 440 nm (Fig. 5A). In the fluorescence scans (Fig. 5B), the emission maximum is reached at 533 nm for the fluorescence emission spectrum, with excitation at 450 nm. The data obtained for the riboflavin isolated from yeast overlapped with the spectrum of the reference riboflavin. In fact, riboflavin identification has been previously determined, where the highest peak was reached at ~ 370 and ~ 440 nm or at ~ 530 nm, for the UV–Vis and fluorescence spectrum, respectively^75,76^.

**Figure 6:**
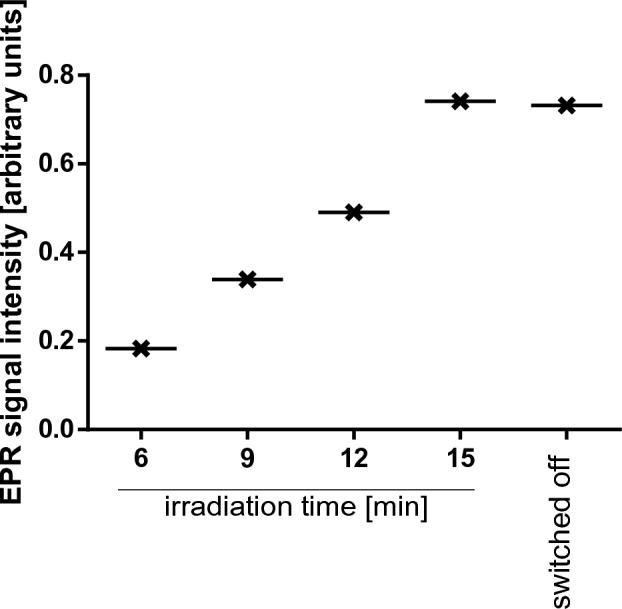
Evolution of the time of the EPR signals (total radicals) during 15 min blue-light irradiation of the RF with the POBN spin trapping agent added.

Oxidative stress plays an important role in homeostasis and disease in most tissues. Reactive oxygen species (ROS) are continuously generated as by-products of normal cellular metabolism^77^. Moreover, ROS are increasingly being recognized as a key component of the bone repair paradigm^78^. RF has been reported to be one of the photosensitizers, which can be activated in certain wavelengths to produce reactive oxygen species with strong oxidation, in order to inactivate pathogenic microorganisms. This unique feature is directly related to its chemical structure, which creates great potential for RF to be used as a mediator to prepare polymer functional materials. The critical structure that determines that RF belongs to the flavin family is a tricyclic structure 7,8-dimethyl-10-alkylisoalloxazine. This essential fragment is responsible for the redox process, subsequent catalytic activity, UV absorption, and photosensitivity^79^. In fact, some studies have been conducted on the role of RF in combination with biopolymers in tissue engineering^80^. The absorbance and fluorescence spectrum of isolated RF may suggest its well behavior as a photosensitizer after irradiation. The next experiment presents an EPR study of the free radicals formed by exposing riboflavin to blue light. POBN has been used as spin-traps for the short-lived free radicals formed during this process. The concentration of radicals after RF irradiation is shown in Fig. 6. Blue-light irradiation increased the initial concentration of radicals in RF, up to the 15 min time of illumination. However, after the light is switched off, the constant level (no further increase) of radicals is observed. This study shows the possibility of using RF as a component of materials for bone regeneration, which may interact after irradiation, resulting in increased radicals. Sel et al. reported on impact of UVA irradiation of riboflavin, which in fact, generated oxygen-dependent hydroxyl radicals^81^. Moreover, it is noticed that vitamin under visible light can also generate reactive oxygen species (ROS), including superoxide anions and singlet oxygen^82^. On the basisof these facts, we can conclude that photoilluminated riboflavin renders the redox status of bacterial cells in a compromised state leading to significant membrane damage, ultimately causing bacterial death. Although we showed the antibacterial potential of composite, the more effective material after its irradiation may have occurred. This study aims to add one more therapeutic dimension to the photoilluminated of composite doped with RF, as it can be effectively employed to targe bacterial biofilms occurring after bioimplantation.

**Figure 7:**
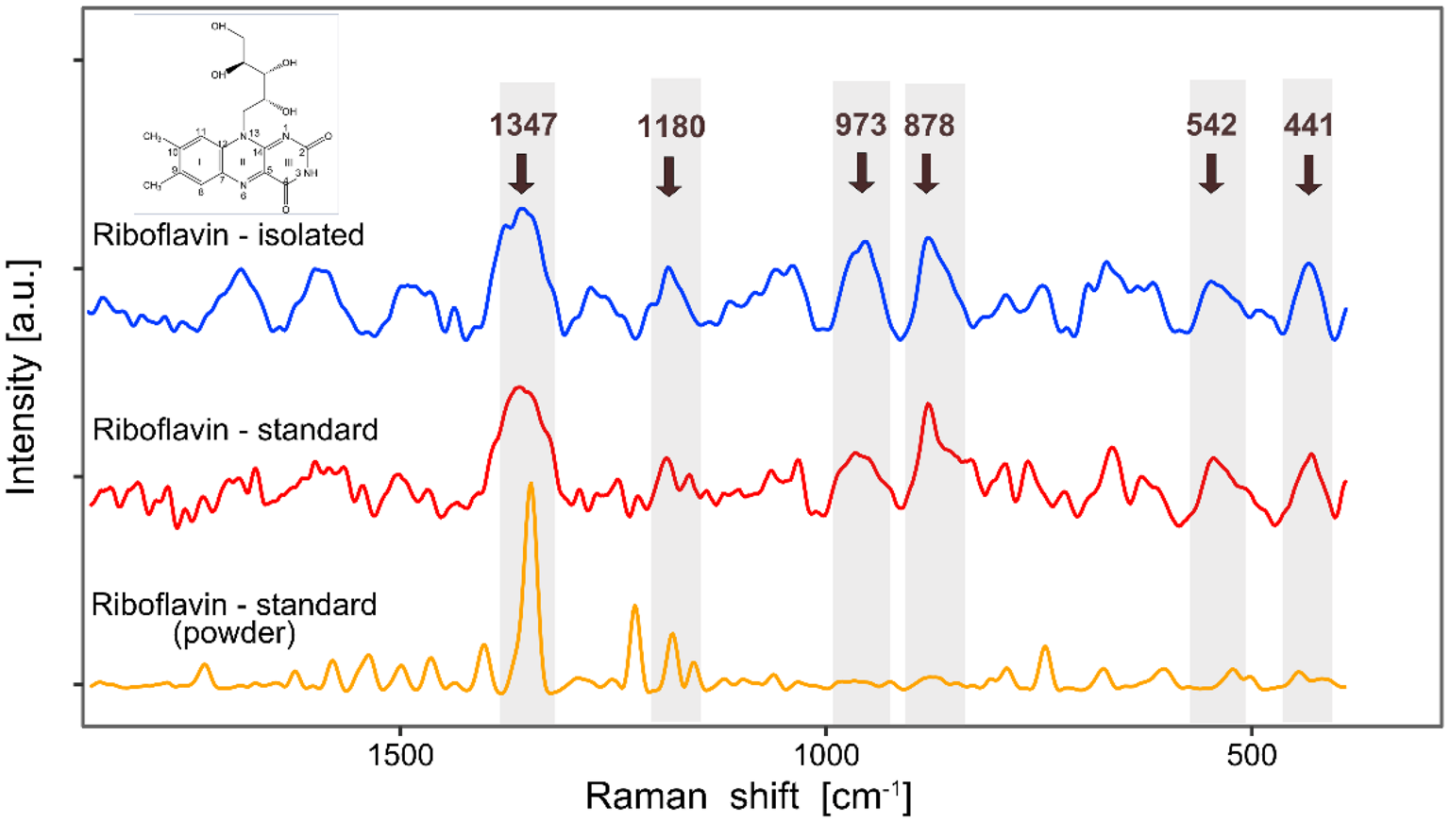
Raman spectra of riboflavin (RF) extracted from yeast (top), the RF standard in solution (middle), and the RF standard in the form of a powder (bottom). The prominent peaks are labelled and shaded grey. The peak around 1347 cm^−1^, assigned mainly to the in-plane vibrations of the isoalloxazine ring, is a unique spectral marker of RF. The inset presents the chemical structure of RF.

The presence of riboflavin in the yeast extract was examined by vibration spectroscopy. Initially applied infrared spectroscopy provided only poor results since the extract deposited on a diamond crystal of the ATR device produced low, and thus noisy, absorbance. This resulted from the fact that only a small amount of dissolved compounds remained on the surface of the ATR crystal after diluent evaporation. More plausible information was achieved using Raman spectroscopy along a microscope device. The spectral characteristics of the extract highly resembled those of the RF standard in solution (Fig. 7). In particular, high conformity in location and shape was observed for the most prominent and specific band around of 1347 cm^−1^, assigned to the in-plane vibrations of the isoalloxazine ring^83^. Moderate absorbances in the low frequency region (around 1180, 973, 878, 542 and 441 cm^−1^), possibly assigned to C–H and O–H deformations vibration of ribityl chain and out-of-plain vibrations of the rings^84,85^ further confirmed the high performance of the extraction process.

**Figure 8:**
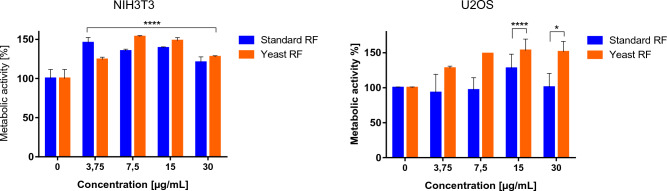
Percentage of metabolic activity of cells after 24 h of exposure to different concentrations of standard and yeast riboflavin. The error bars indicate the standard deviation. Data analysis was carried out by two-way ANOVA and averaged from three replicates (n = 3).

Riboflavin is a well-known component of flavo-coenzymes important in numerous reactions. But other biological properties have also been ascribed to riboflavin. Among them, its additive effect on osteoblast differentiation of MC3T3-E1 cells seems promising and may be a therapeutic approach to the treatment of osteoporosis^30^. Moreover, the development of biomaterials with unique features has recently attracted great attention for bone regeneration, wound healing, and medical purposes^86^. Thus, we also performed experiments to assess the biological activity of RF as a future component of the composite material. The potential effect of RF on cell metabolic activity was tested using the MTT assay, which measures cell mitochondrial activity through the NAD(P)H-dependent cellular oxidoreductase enzyme^87^. The metabolic activity of various concentrations of riboflavin in two different cell lines: mouse embryonic fibroblasts (NIH 3T3) and human osteosarcoma (U-2OS) is shown in Fig. 8. We found that treatment with riboflavin significantly increased cell metabolic activity, especially when exposed to the isolated vitamin from a microorganism. The results show that this metabolite does not have a toxic impact on the tested cell lines. Indeed, our data are in line with the Chaves Nato et al. who reported on riboflavin and its irradiated version, which did not affect osteoblast viability^30^. Moreover, the protective effect of riboflavin as a component of other materials has also been suggested. Xizhe Li et al. manufactured ultrasmall riboflavin-protected silver nanoclusters (RF@AgNCs) that can effectively kill or suppress pathogen growth. At the same time, they were found to be non-toxic to human red blood cells and mammalian cells^88^.

**Figure 9:**
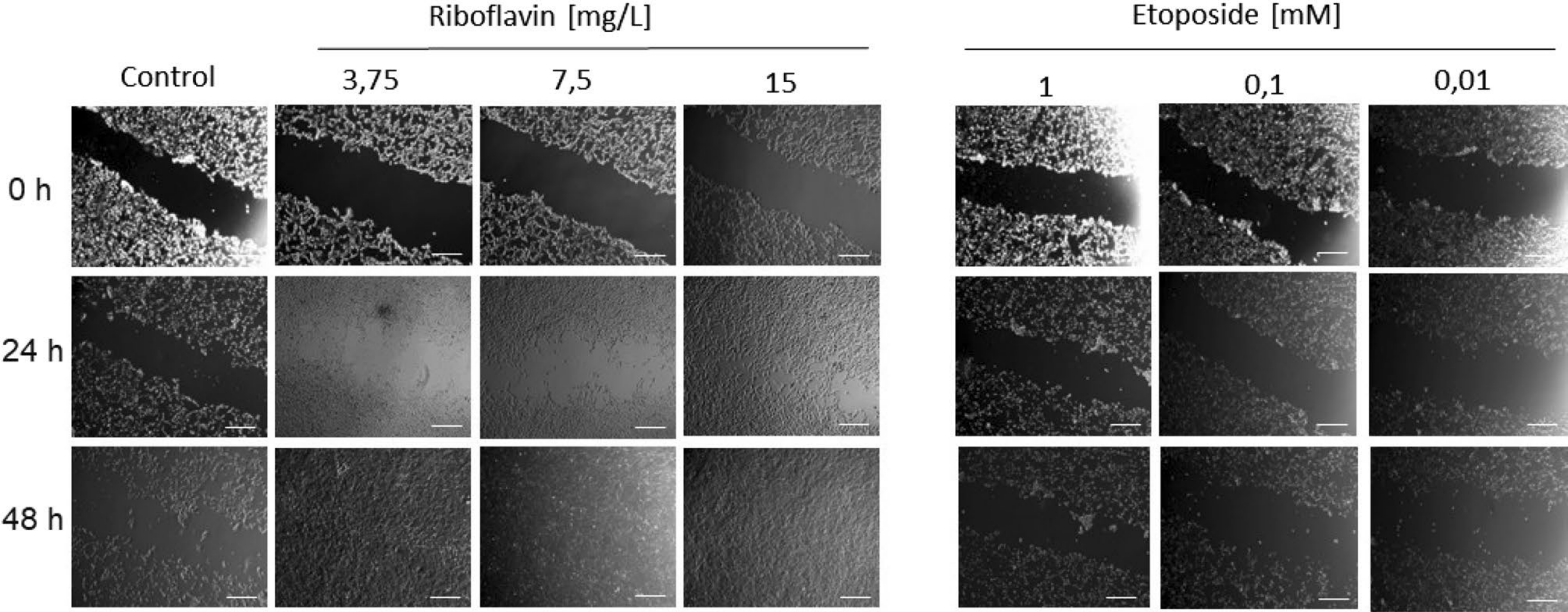
Microscope observation of scratch closure after treatment of mouse fibroblasts (NIH3T3) and etoposide with riboflavin. Arrows indicate the scratched area, scale bar 200 µm. Time zero corresponds to the addition of RF.

Many authors usually report on the cytotoxicity or biocompatibility of the compound tested with respect to cells based on the only one method used for the analysis. Therefore, in this study, either the MTT assay or wound healing assay have been performed to evaluate the metabolic activity or the motility capacities of the cells, respectively. Cell mobility plays a crucial role in many physiological and pathological processes. Cell migration occurs during embryo development, wound healing, or immune response. The motility capacities has also been implicated in many diseases such as cancer or inflammation^89^. Therefore, cell mobility could be used as a parameter to assess the physiological state of the cell^42,90^. To evaluate the migration response of NIH3T3, cells were exposed to different concentrations of RF and allowed to migrate for 48 h (Fig. 9). Etoposide was used as chemical that exhibits a negative impact on the wound-healing ability of the cell^91^.

After 48 h of exposure of cells to riboflavin, the scratch closure of NIH3T3 cells was close to 100%, for each concentrations tested (Fig. 9). Here, the etoposide acts as a chemical which inhibited the migration of the cells. On the basis of these two biological experiments, we concluded that riboflavin had no negative effect on cell migration and its metabolic activity. Furthermore, it may play a role as a compound that activates cell proliferation.

## Conclusions

Developing non-cytotoxic composite materials with unique additional functions is crucial for bone regeneration and tissue engineering fields. The present study demonstrated the effectiveness multifunctional composite. The material can be prepared into scaffold using chitosan as a matrix and hydroxyapatite and riboflavin as a phase filler. The porous surface morphology and possible interlinkage between the components, after doping of the pure chitosan, were confirmed through the SEM and FTIR analysis, respectively. Compared to existing technologies for the manufacture of the chiotsan/HAP scaffold, doping it with riboflavin showed the novel features; i.e. antimicrobial potential and antioxidant activity. Moreover, the non-cytotoxic RF significantly enhanced cell proliferation and migration, and is light-activated, producing the radicals. Therefore, this study demonstrated the new material that could be useful for bone tissue regeneration.

## Data Availability

The data sets used and/or analysed during the current study available from the corresponding author on reasonable request.
